# Quantifiable Measures of Abdominal Wall Motion for Quality Assessment of Cine-MRI Slices in Detection of Abdominal Adhesions

**DOI:** 10.3390/jimaging9050092

**Published:** 2023-04-30

**Authors:** Bastiaan A. W. van den Beukel, Bram de Wilde, Frank Joosten, Harry van Goor, Wulphert Venderink, Henkjan J. Huisman, Richard P. G. ten Broek

**Affiliations:** 1 Department of Surgery, Radboud University Medical Center, 6525 GA Nijmegen, The Netherlands; 2 Department of Radiology and Nuclear Medicine, Radboud University Medical Center, 6525 GA Nijmegen, The Netherlands; 3 Department of Radiology and Nuclear Medicine, Hospital Rijnstate Arnhem, 6815 AD Arnhem, The Netherlands

**Keywords:** adhesions, cine-MRI, diagnostic quality, biomarker, algorithm

## Abstract

Abdominal adhesions present a diagnostic challenge, and classic imaging modalities can miss their presence. Cine-MRI, which records visceral sliding during patient-controlled breathing, has proven useful in detecting and mapping adhesions. However, patient movements can affect the accuracy of these images, despite there being no standardized algorithm for defining sufficiently high-quality images. This study aims to develop a biomarker for patient movements and determine which patient-related factors influence movement during cine-MRI. Included patients underwent cine-MRI to detect adhesions for chronic abdominal complaints, data were collected from electronic patient files and radiologic reports. Ninety slices of cine-MRI were assessed for quality, using a five-point scale to quantify amplitude, frequency, and slope, from which an image-processing algorithm was developed. The biomarkers closely correlated with qualitative assessments, with an amplitude of 6.5 mm used to distinguish between sufficient and insufficient-quality slices. In multivariable analysis, the amplitude of movement was influenced by age, sex, length, and the presence of a stoma. Unfortunately, no factor was changeable. Strategies for mitigating their impact may be challenging. This study highlights the utility of the developed biomarker in evaluating image quality and providing useful feedback for clinicians. Future studies could improve diagnostic quality by implementing automated quality criteria during cine-MRI.

## 1. Introduction

Cine-MRI has been demonstrated to be a useful imaging modality for diagnosing and mapping adhesions in patients with chronic abdominal complaints after surgery [1,2]. Adhesions are a form of internal scar tissue that forms after 90% of open surgeries and 70% of laparoscopic surgeries in the abdomen [3]. Adhesions are the etiology of pain in 25–50% of patients with chronic post-operative abdominal or pelvic pain [4,5]. Radiological diagnosis of adhesions is challenging because adhesive tissue lacks a mass that can be visualized as a lesion using conventional imaging modalities. Therefore, diagnostic laparoscopies needed to be performed in patients with suspected adhesions, resulting in significant numbers of negative laparoscopies (20%) as well as complications such as bowel injuries (7%) [6,7]. Using dynamic imaging modalities such as cine-MRI or ultrasound, adhesions can now be detected by assessing visceral slides [8,9]. Visceral slide refers to the smooth sliding motion that occurs between the abdominal contents and the ventral, lateral, and dorsal walls of the abdominal cavity during respiratory movements [10]. During respiration, the abdominal cavity deforms, causing an upwards motion of organs during the expiration [9]. The absence of a normal visceral slide pattern indicates a potential adhesion. Mapping of adhesions can guide therapy in patients with chronic symptoms of adhesions, resulting in a lower risk of negative laparoscopy and injuries, and improving the long-term efficacy of adhesiolysis to reduce pain [1,6,8]. 

A deep and smooth abdominal breathing movement made by the patient is required to obtain an optimal quality of visceral slide and enhance the reliability of mapping of adhesions. Performing such deep abdominal breathing movements appears to be difficult for a relatively large number of patients. A previous study has shown that approximately 50% of the slices made during the cine-MRI scan, had a suboptimal motion of the viscera due to inadequate breathing and squeezing, using a subjective scale [11]. Despite the relatively large number of slices of suboptimal diagnostic quality, the overall sensitivity and specificity for the diagnosis of adhesions on cine-MRI remain high at a patient level. However, slices with insufficient motion do seem to impact the mapping of adhesions which is crucial for operative risk assessment and planning. Factors that might impact movement are patients’ compliance with movement instructions, quality of the instruction, patients’ capacity to understand and reproduce movements, and other patient-related factors such as having a stoma, obesity, or health conditions that impair movement [12,13,14]. Quality of instruction might be improved by the use of video instruction, as research in other fields has shown that visual instructions can improve the understanding of instructions related to motor skills [15].

To improve the quality of cine-MRI, there is a need for methods to assess, control, and improve the quality of movements [16]. Obtaining cine-MRI slices results in a short film, from which several biomarkers can be measured including the amplitude, frequency, and slope of movement. We hypothesize that these biomarkers can be used for the evaluation of the quality of movement on cine-MRI. Further, insight into patient-related factors that might impact movement on cine-MRI could be useful to improve quality.

This study aims to develop a quantifiable biomarker for movement on cine-MRI that correlates with expert radiological quality grading of the cine-MRI scan. Second, we assessed the impact of patient-related factors on movement on cine-MRI. 

## 2. Materials and Methods

### 2.1. Study Design and Patients

All patients who had one or more cine-MRI scans between 2012 and 2021 in the diagnostic work-up for chronic abdominal pain and adhesive small bowel obstruction, were included. All cine-MRI scans were made at one hospital (Rijnstate) with a 1.5 Tesla MRI scanner under the supervision of experienced radiology technicians and readers. Patients were admitted for cine-MRI scans by the outpatient clinics of the Radboud University Medical Center in Nijmegen and the Rijnstate Hospital in Arnhem. In 2019 a video instruction for patients was introduced in preparation for patients undergoing cine-MRI from Radboud University Medical Center. Imaging data from cine-MRI scans and data from electronic files were retrospectively collected. A waiver was obtained from the medical ethical committee of the Radboud university medical center for this study, according to Dutch law (METC registration number: 19082). Data were managed according to FAIR principles. 

Data from electronic patient records and radiology reports were recorded and baseline factors that might affect the quality of movement during cine-MRI were extracted. Factors were selected based on the qualitative assessment of the radiology reports for remarks on factors that impacted the quality of the scan or caused artifacts, and the suggestions from experienced readers (FJ, WV, RB) [14].

### 2.2. Technique of Cine-MRI 

Cine-MRI images of a single 1.5-Tesla scanner (Siemens medical solutions, Erlangen, Germany) were obtained using the protocol first described by Lienemann et al. [2,17]. For the cine-MRI sequence, we used a true-FISP scan, with echo and relaxation times of 1.53 and 3.66 ms, a flip angle of 60°, a matrix size of 192 × 256, slice thickness of 5 mm, 2.3 frames per second and 30 frames per imaged slice. No contrast or specific preparation other than movement instructions were applied. Typically, five to six sagittal slices were acquired at the midline, left/right paramedian, and left/right lateral at the ascending/descending colon. 

### 2.3. Subjective Quality Grading 

A random selection of 90 cine-MRI slices from 30 original studies was taken from the period 2015–2018 (Supplementary Materials; Figure S1). This period was selected to exclude the initial learning curve of technicians and to select cases prior to the introduction of video instruction. These slices were reviewed for quality grading by two experienced readers independently. One reader is an expert radiologist with 30 years of experience, and the other is a clinical expert involved in adhesion care since 2012. Both have reviewed hundreds of cine-MRI scans. Reviewers graded overall quality and quality for the biomarker amplitude, frequency, and slope of the motion on a 5-point scale (0 = poor, 1 = insufficient, 2 = sufficient, 3 = good, 4 = excellent). A score of 0 or 1 was considered insufficient. The grading was performed using an interactive online reader study platform on https://grand-challenge.org, accessed on 15 February 2023. Each observer reviewed the scans between April and November 2021, independently in a workstation of their own preference.

Intraclass correlation Coefficient (ICC) was measured for the overall quality grading and the three biomarkers (amplitude, frequency, and slope). An ICC above 0.60 was considered good reliability [18]. Interobserver reliability was measured with the intraclass correlation coefficient, correlation of the biomarkers, and overall quality of cine-MRI was measured with Cronbach’s alpha of coherence. 

### 2.4. Development of Image Processing Algorithm for Biomarkers 

For quantitative analysis, an algorithm was developed to measure the displacement of the anterior abdominal wall. The algorithm tracks the displacement of each pixel along the anterior abdominal wall over time. For each time point, a single displacement value is obtained by averaging the displacement of all pixels. This results in a displacement curve, from which amplitude, frequency, and slope can be determined. The algorithm automatically recognizes and reports two situations in which it is not deployable: first when a patient is very obese and a part of the pixels falls outside the field of view, and second when severe artifacts are present. The amplitude is the difference between the maximum and minimum displacement in millimeters, the frequency is estimated by counting the number of minima and maxima in the curve expressed in Hertz and the slope is determined by taking the maximum of the derivative of the curve in millimeters per second. To test the validity of the algorithm to quantify biomarkers for cine-MRI, the results were compared to manual measurements of amplitude and frequency biomarkers. These measurements were taken in a DICOM viewer (ITK-SNAP version 3.8) on a random selection of 22 cine-MRI slices by a researcher independent of the qualitative gradings by the experienced readers [19]. We did not perform manual measurement of slope, because manual assessment of slope was impractical and time-consuming. Moreover, the biomarker for slope was found to be correlate strongly with amplitude, and was excluded from further analysis. For this reason manually measurement of slope was also deemed to be of minor interest. 

### 2.5. Correlation between Biomarker and Quality Grading

To assess the correlation between the biomarker as measured by the algorithm and subjective quality, the rounded-down average score of the two readers of the quality grading was used. The correlation between the biomarkers and subjective quality gradings was assessed using linear regression or the Mann–Whitney U-test if data were not normally distributed. A *p*-value < 0.05 was considered significant. The distribution of continuous variables was assessed using stem-and-leaf and histogram plots. 

To establish an automatic quality criterion using the algorithm-based biomarkers, the 90 graded slices from 30 studies were randomly divided into a training set (60 slices from 20 studies) and a test set (30 slices from 10 studies). Receiver Operating Characteristic (ROC) analysis was used on the training set to determine the optimal threshold for each biomarker to discriminate between cine-MRI scans of sufficient (scores 2, 3, and 4) and insufficient (scores 0, 1) quality and to assess individual discriminatory power concerning subjective quality scoring. Confidence intervals (95%) are estimated with bootstrapping, using 10000 iterations to reliably estimate the confidence interval on the Area Under the Curve [20]. After establishing the quality criterion based on the biomarkers, the remaining 10 studies were used as a separate test set to validate performance using sensitivity and specificity. The results were visualized using scatterplots. 

### 2.6. Impact of Patient-Related Factors on Movement on Cine-MRI 

Factors of interest extracted from electronic patient records and radiology reports by experienced readers were sex, age, having a stoma, presence of a ventral hernia, gastric bypass in history, adhesions on cine-MRI, length, weight, BMI, artifacts, and use of video instruction in preparation for cine-MRI [14]. All patients in this study received a leaflet with general MRI instructions in preparation for the cine-MRI. During cine-MRI, all patients also received spoken instructions from technicians that are experienced in obtaining cine-MRI scans. In January 2019 we implemented a video to explain movement instructions for cine-MRI in the outpatient clinic of Radboud University Medical Center. Patients referred for cine-MRI from the Rijnstate Hospital continued to receive a leaflet with written instructions after 2019. The video was evaluated by laymen and radiology technicians before implementation. The video includes a voice-over in Dutch explaining the movements. The video instruction is available online and easy to play on any electronic device [https://youtu.be/qi3GoaKsXuI], accessed on 1 December 2022. 

For evaluation of the impact of patient-related factors on the movement on cine-MRI, we made use of the newly developed automatic quality criterion as described above. We assessed the impact of each factor on the percentage and number of slices per scan with sufficient movement as measured by the threshold of the biomarker. Univariate and multivariate linear regression analyses were performed to identify factors influencing the percentage and number of slices with sufficient movement. Risk factors with *p* ≤ 0.20 in univariate were selected as candidate factors for multivariate analysis. In multivariate analysis, a stepwise backward selection procedure was used with a *p*-entry ≤ 0.20 and *p*-stay ≤ 0.10. All statistical analyses were performed using SPSS version 28∙0 (Armonk, NY, USA: IBM Corp).

## 3. Results

Between February 2012 and February 2021, 560 cine-MRI scans in 527 patients were included. The prevalence of patient-related factors has been listed in the baseline table (Table 1). Included patients were predominantly female (75.4%), and the mean age was 50.7 (±14.0 y). Post-operative anatomical changes that might impact abdominal wall movement were present in 94 (16.8%) patients. Of these patients, 49 (52.1%) patients had a stoma, 21 (22.4%) patients had a ventral abdominal wall hernia, and 24 (25.5%) had a gastric bypass. 

### 3.1. Subjective Quality Grading 

Of the 90 slices analyzed, 52 (57.8%) slices were graded sufficiently for diagnosing adhesions after averaging the scores of both readers. Interobserver reliability for the overall quality of the scans on a 5-point grading scale was high with an Intraclass Correlation Coefficient (ICC) for consistency of 0.729 (95% CI 0.590–0.821; *p* < 0.001) and an ICC for absolute agreement of 0.732 (95% CI 0.593–0.824; *p* < 0.001). The ICC for consistency between both reviewers for the three biomarkers amplitude, slope, and frequency was 0.728 (95% CI 0.586–0.821; *p* < 0.001), 0.682 (95% CI 0.516–0.790; *p* < 0.001) and 0.632 (95% CI 0.442–0.758; *p* < 0.001) respectively. The correlation between the score for amplitude, slope, and frequency and the overall quality grade of cine-MRI was high with a Cronbach’s Alpha of 0.883 (Supplementary Materials; Table S1).

### 3.2. Biomarker and Manual Measurements 

**Figure 1:**
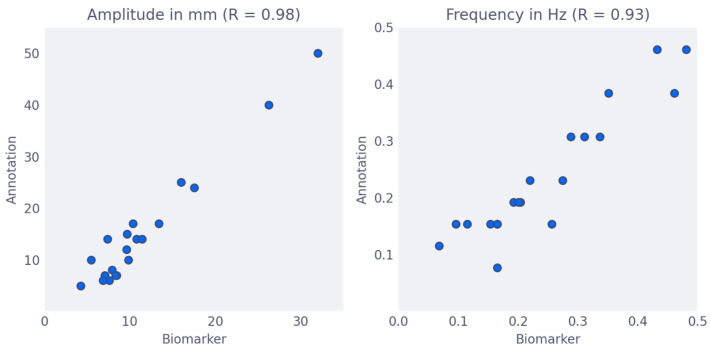
Comparison of biomarkers to manual annotation. Comparison of the biomarkers as calculated by the algorithm (y-axis) to manual annotation (x-axis). (**Left**) The movement amplitude was measured in mm with a Pearson’s Correlation Coefficient (R) of 0.98, (**Right**) the movement frequency was measured in Hz with a Pearson’s Correlation Coefficient (R) of 0.93.

Two slices of the 22 randomly selected slices for manual measurement of movement could not be processed by the algorithm, because one featured a very obese patient and one had severe artifacts. The amplitude and frequency as measured by the algorithm almost perfectly correlated with manual measurements of these parameters [Figure 1], with correlation coefficients of 0.98 and 0.93, respectively. Amplitude values of the algorithm biomarker were consistently lower than the manually measured amplitude by a factor of 1.4. This is explained by the fact that the algorithm averages the displacement along the entire anterior wall, which is impractical for manual measurement. Instead, manual measurement was performed at the point of maximal displacement. The slope was not measured manually, and values estimated by the algorithm turned out to be strongly correlated (R = 0.88) to the amplitude. It was therefore not further considered in the quality criterion (Supplementary Materials).

### 3.3. Association between Biomarker and Subjective Quality 

**Figure 2:**
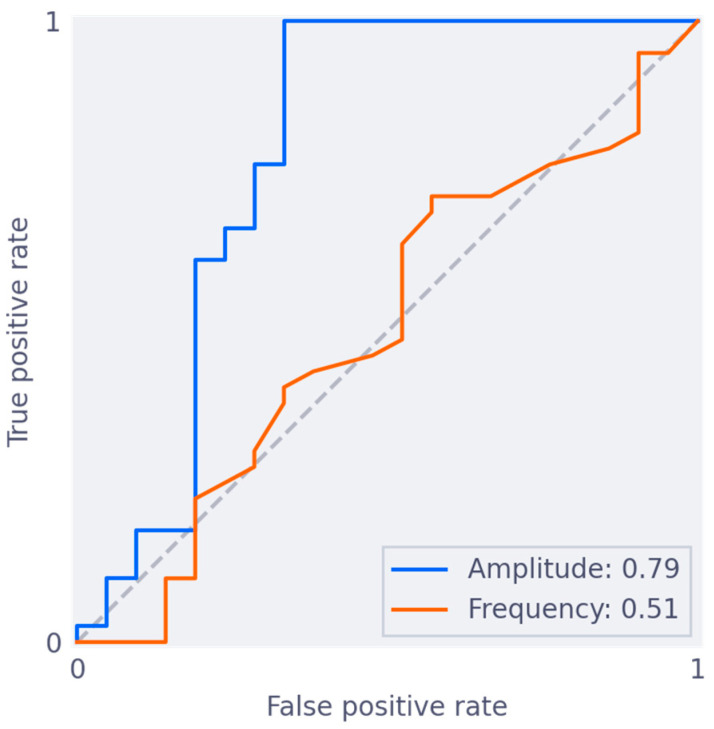
ROC curves on train set for classification of insufficient vs. sufficient slices. ROC curves on the training set for classification of insufficient (0, 1) vs. sufficient (2, 3, 4) slices, using only the Amplitude biomarker (blue) or only the Frequency biomarker (orange). The Amplitude biomarker has an AUC of 0.79, and the Frequency biomarker has an AUC of 0.51.

**Figure 3:**
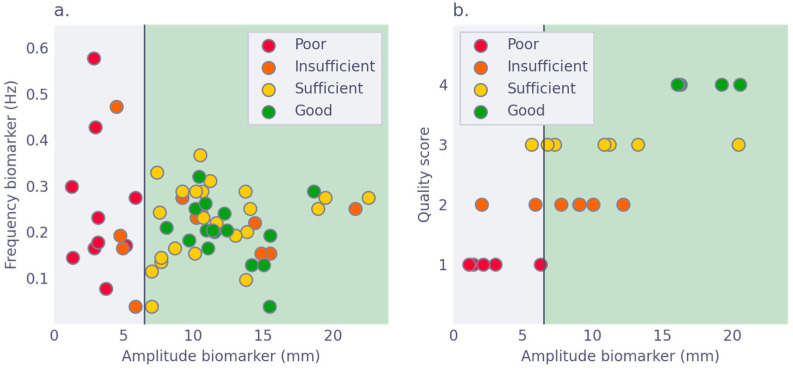
Quality criterion differentiates between sufficient and insufficient quality. (**a**) The Frequency and Amplitude biomarker for each case in the 30-slice test set, where the colors of the circles, red to green, correspond to the subjective quality score, Poor to Good. The vertical line visualizes the amplitude criterion. (**b**) The same plot as (**a**), but with the subjective quality score on the y-axis.

Amplitude and frequency were significantly correlated to the qualitative grading of overall quality, and qualitative grading per respective biomarker (Supplementary Materials; Table S2). The curves of ROC analysis are presented in Figure 2. The criterion for amplitude was able to discriminate insufficient and sufficient slices, with an area under the ROC curve (AUC) of 0.79 (95% CI 0.61, 0.92). Taking an amplitude of at least 6.5 mm as a cut-off for sufficient quality, resulted in 100% sensitivity and 67% specificity on the training set of 60 slices. The criterion for frequency was unable to discriminate insufficiently from sufficient scans, with an AUC of 0.51 (95% CI 0.34, 0.66). A combined cut-off point, combining both amplitude and frequency, did not result in better performance. Further analysis was therefore performed with a criterion based on amplitude alone, using the 6.5 mm cut-off. The performance of this criterion is visualized in Figure 3. 

To validate the quality criterion based on amplitude, we tested the 6.5 mm cut-off on a separate set of 30 slices. The algorithm was unable to process 6 slices because they featured obese patients in whom part of the pixels fell outside the visualized field. The criterion resulted in a sensitivity of 92% and specificity of 58% for the detection of slices of sufficient quality on the remaining 24 slices. The criterion is visualized in Figure 3. Only one slice of sufficient quality did not pass the quality criterion. Further, all slices that received the lowest quality grade (poor) were detected as insufficient by this criterion in both the training and test set (Figure 3). 

Overall, in this selection of 90 slices, there were discrepancies between the automatic quality criterion and results of qualitative grading in 13 (14.4%) slices; predominantly (*n* = 12), these were false positive results. Reviewing the 12 false positive slices from 8 patients revealed artifacts that could explain the discrepancy in 3 slices from 2 patients. Further, disrupted anatomy from the stoma and gastric bypass might also have impacted the quality of the cine-MRI scan in three other slices from two patients. Although these factors do not always seem to interfere with quality, in these two patients these factors were also mentioned in the original radiological report to have impacted the readability of the cine-MRI scan. For the remaining seven slices, the discrepancy could not be attributed to an apparent cause.

### 3.4. Patient-Related Factors and Movement on Cine-MRI 

From a total of 560 cine-MRI scans, 3535 slices were eligible for analysis. The algorithm was unable to process 124 slices, because of obesity in 113 slices and severe artifacts in 11 slices. The remaining 3411 slices were included in the analysis, of which 2468 (72.4%) passed the quality criterion of an amplitude of 6.5 mm. In 40 patients (7.1%) none of the slices reached the 6.5 mm threshold, and in 226 (40.4%) all slices reached the threshold. 

Results of uni- and multivariate analysis on the impact of patient factors and video instruction on the percentage of slices with sufficient quality per patient on cine-MRI can be found in Table 2. In multivariate analysis, higher age, higher length, and male sex were independently associated with a higher percentage of slices with sufficient quality. The presence of a stoma was independently associated with a lower percentage of slices with sufficient quality (R^2^ = −0.178, *p* = 0.002). All other risk factors were excluded from the multivariate analysis. 

Results of uni- and multivariate analysis on the impact of patient factors and video instruction on the number of slices with sufficient quality per patient on cine-MRI can be found in Table 3. Results from multivariable analysis were comparable to the results of the analysis of the percentage of sufficient slices per patient. In this analysis, however, the number of slices made was also independently associated with an increase in the number of slices with sufficient movement. Video instruction was removed by backward selection in our multivariate analysis.

## 4. Discussion

The amplitude of movement was found to be a reliable biomarker for the quality of movement on cine-MRI. This finding supports the hypothesis that sufficient patient movement is key to obtaining optimal diagnostic results. Amplitude can be measured fast and reliably using the algorithm. A cutoff of 6.5 mm of abdominal wall displacement was indicative of sufficient quality of movement. Patient factors that positively impact the amplitude of movement on cine-MRI were length, male sex, and higher age. The presence of a stoma negatively impacted amplitude. None of these patient factors were amendable for improvement.

Cine-MRI has been demonstrated to be a useful tool in the diagnosis of adhesions, especially in patients with chronic postoperative pain [1,8,17]. Improved patient selection by the implementation of cine-MRI results in a lower number of negative diagnostic laparoscopies, and reduces the risk of adhesiolysis-related injuries during reoperation for chronic pain [1]. Cine-MRI is also useful in the work-up of patients with recurrent small bowel obstruction [21]. In a recent systematic review, the overall accuracy of cine-MRI to detect adhesions on a patient level was 76–100% [6]. Nevertheless, concerns with the implementation of cine-MRI in quality control remain. Improved quality of the cine-MRI is needed to provide a more accurate mapping of the location of adhesions. Mapping of adhesions is most useful for preoperative planning [3,22,23]. Randall et al. previously described that 50% of slices subjectively had insufficient motion [11]. Second, there is relatively little experience with cine-MRI and high inter-rater variability [24]. To improve the radiological reading of cine-MRI attempts have been made to develop computer-aided detection. Early experiments have been performed using an image registration-based technique called the ‘shearogram’. Although this technique showed some promising first results, it also comes with some important limitations including the inability to detect adhesions between organs [11,12]. Currently, AI and radiomics are increasingly being used to develop computer-aided detection systems [25]. Most of these AI-based computer-aided detection systems, however, use still images, and computer-aided detection for moving pictures in health is still in its early phase [26].

In this study, we focused on the movement aspect of cine-MRI as a potential factor for future quality improvement. A limitation is the lack of a gold standard to define the quality of cine-MRI. The current assessment is based on the judgment of experienced readers. However, the high inter-observer reliability between experienced readers on both the overall quality and several aspects of movement, shows that there is validity to these judgments of quality. As a tertiary referral center for adhesion-related complaints, we have a relatively high volume of cine-MRI scans and experienced readers. Such experienced readers on cine-MRI might not be available in most other centers.

The strong correlation between the biomarker for amplitude and quality, as graded by expert readers, is promising. We developed an algorithm-based criterion for amplitude that can be measured reliably and fast. This algorithm had high sensitivity; however, the specificity was still relatively low. Ideally, a higher specificity for slices with insufficient quality should be achieved. The relatively low specificity might be explained by the fact that movement is a surrogate marker for overall quality. In approximately half of the slices where there was a discrepancy between subjective graded quality and biomarker results, non-amendable factors such as artifacts or disrupted anatomy contributed to the discrepancy. In only a small group of patients the criterion for amplitude results in an inexplainable incorrect judgment that could be improved by redoing the same slice. Moreover, the algorithm seemed to recognize all cases in which movement was poor. Therefore, we consider this biomarker to be useful even though specificity needs further improvement.

Patient-related factors were selected based on remarks on quality in the radiology reports, and the suggestion from experienced readers [14]. In previous literature, the importance of sufficient induced visceral slide has been mentioned as a key factor in the diagnosis of adhesions. However, methods for measuring movement were unclear, and no assessment of patients-related factors on movement had been made [23].

A few patient-related factors were demonstrated to impact movement on cine-MRI, however, none of these were amendable for improvement. Based on the ample body of literature on video instruction for motor skills, we expected that video instruction would improve the quality of movement on cine-MRI. This was not the case. Potentially the experienced team of operators is sufficiently able to provide clear instructions both during and after the scan. In that case, video instruction might have a greater impact on the quality of movement in centers that are earlier in the implementation of Cine-MRI. Another explanation for the lack of improvement seen with the introduction of video instructions is that some patients have great difficulty in performing deep abdominal breathing. It is known from the literature that a relatively large number of people by default perform chest breathing [27]. For some patients, a video tutorial alone may not be adequate in guiding them through taking deep abdominal breaths [28].

In our results, in only 7% of patients, none of the slices had sufficient movement. In most cases, patients had mixed results with slices of both sufficient and insufficient movement in their cine-MRI series. These results seem to indicate that most patients are capable of making sufficient deep abdominal breathing and that results are amenable for further improvement. Real-life training might be more effective than video instruction to improve movement [29]. Important drawbacks are that real-life training is time and cost-intensive and does not assure that patients will make good deep abdominal breathing in every slice.

Providing direct biofeedback during the scans might be a more promising and cost-effective approach to improve movement. Biofeedback might be provided by the operator of the MRI, as we demonstrated that experienced were able to score amplitude on a 5-point Likert scale. However, this requires quite a lot of experience with the reading of cine-MRI. Manual measurement of amplitude is highly elaborate. Implementation of the biomarker could overcome these issues. During the scanning, the algorithm could provide direct biofeedback on amplitude, and provide advice about slices to redo to obtain better results. Future studies are required to make the algorithm available for on-the-fly use during the scanning procedure and demonstrate if this will impact the overall quality of the scan. Future studies should also explore if additional biomarkers could further improve the predictive value of the marker. Currently, the biomarker is calculated from the outward movement of the anterior abdominal wall. Possibly, also the upward movement of the diaphragm should be considered.

The development of such biomarkers could also facilitate a wider implementation of cine-MRI in diagnosis adhesions in relation to chronic abdominal pain. Higher quality of cine-MRI directly improves the diagnosis of adhesions and will improve shared decision-making for adhesiolysis. Despite the promising studies on diagnostic accuracy, and the clinical application of cine-MRI in chronic adhesion-related pain and to minimize iatrogenic injuries during reoperation, implementation of cine-MRI remains low. Technically, implementation of cine-MRI seems feasible as the requirements of a 1.5 Tesla MRI and packages for cine functions are becoming ever more prevalent. The learning curve and experience required to obtain sufficient quality cine-MRIs and reporting seem to be the most important hurdles for implementation [30]. Algorithms for quality improvement and computer-aided detection could therefore support clinical centers starting with implementation.

## 5. Conclusions

The quality of cine-MRI is strongly affected by the movement of the anterior abdominal wall. We have developed an automatic quality criterion that uses the amplitude of this movement to accurately determine whether a scan is sufficient or insufficient for reading. Patient factors that positively impact the amplitude of movement on cine-MRI were length, male sex, and higher age. The presence of a stoma negatively impacted amplitude. This newly developed quality criterion can be used in future cine-MRI studies to optimize the movement of the anterior abdominal wall, and thereby the quality of the scan.

## Figures and Tables

**Table 1:** Baseline Characteristics.

Factors	N (%)/(±SD)/(Range)
Male	138 (24.7%)
Female	422 (75.3%)
Age (in years)	50.7 (±14.0)
BMI (kg/m^2^)	26.0 (±5.2)
Stoma	49 (8.8%)
Ventral abdominal wall hernia	21 (3.2%)
Adhesions	378 (68.9%)
Gastric bypass	24 (4.3%)
Artifacts	6 (1.1%)
Length (in cm)	169.9 (±9.1)
Weight (in kg)	75.3 (±17.6)
Video instruction	134 (23.9%)
Number of slices per patient	6 (4–18)
Number of sufficient slices per patient	5 (0–16)
Percentage of sufficient slices per patient	60.1 (±38.7)
Total number of unreadable slices	124 (3.5%)
Total number of slices	3535 (100%)
Number of scans	560
N (%) or Median (range) or mean (±SD)	

**Table 2:** Uni- and multivariate analysis factors affecting the percentage of sufficient Cine-MRI.

Factor	Univariate Regression Coefficient	95% CI	*p*-Value	Multivariate Regression Coefficient	95% CI	*p*-Value
Sex (male)	0.020	−0.047–0.088	0.550	0.110	0.015–0.204	0.023
Age (for each year)	0.002	0.000–0.004	0.052	0.003	0.001–0.006	0.005
Stoma (yes)	−0.096	−0.198–0.006	0.064	−0.178	−0.292–−0.063	0.002
Abdominal wall hernia (yes)	−0.056	−0.208–0.096	0.469	-	-	-
Gastric Bypass (yes)	−0.010	−0.153–0.133	0.892	-	-	-
Adhesions (yes)	−0.005	−0.068–0.058	0.867	-	-	-
Artifacts (yes)	−0.194	−0.475–0.087	0.175	-	-	-
BMI (for each kg/m^2^)	0.001	−0.006–0.007	0.848	-	-	-
Length (for each cm)	0.005	0.001–0.009	0.007	0.009	0.004–0.013	0.001
Weight (for each Kg)	0.001	−0.001–0.003	0.163	-	-	-
Video instruction (yes)	−0.011	−0.079–0.057	0.752	-	-	-
Number of slices per patient	−0.004	−0.014–0.021	0.675	-	-	-

**Table 3:** Uni- and multivariate analysis: Patient-related factors affecting the number of sufficient cine-MRI slices.

Factor	Univariate Regression Coefficient	95% CI	*p*-Value	Multivariate Regression Coefficient	95% CI	*p*-Value
Sex (male)	−0.091	−0.549–0.367	0.697	0.627	0.062–1.191	0.030
Age (for each year)	0.012	−0.002–0.026	0.098	0.014	0.000−0.028	0.044
Stoma (yes)	−0.890	−1.584–−0.195	0.012	−1.203	−1.887–−0.519	0.001
Abdominal wall hernia (yes)	−0.568	−1.605–0.470	0.283	-	-	-
Gastric Bypass (yes)	−0.117	−1.091–0.857	0.814	-	-	-
Adhesions (yes)	0.128	−0.230–0.558	0.558	-	-	-
Artifacts (yes)	−1.587	−3.499–0.325	0.104	-	-	-
BMI (for each kg/m^2^)	0.006	−0.038–0.050	0.786	-	-	-
Length (for each cm)	0.037	0.012–0.061	0.004	0.044	0.017–0.070	0.001
Weight (for each kg)	0.010	−0.003–0.023	0.122	-	-	-
Video instruction (yes)	0.804	0.346–1.261	0.001	-	-	-
Number of slices per patient	0.750	0.649–0.852	0.001	0.729	0.612–0.846	0.001

## Data Availability

The data presented in this study are available on request from the corresponding author. The data are not publicly available due to privacy and property reasons.

## Supplementary Materials

The following supporting information can be downloaded at: https://www.mdpi.com/article/10.3390/jimaging9050092/s1, Figure S1: Flowchart Quality Criterion; Figure S2: Slope Biomarker; Table S1: Correlation of quality assessment between reviewers; Table S2: Correlation of biomarker and semi-quantitative quality assessment; Table S3: Biomarker vs Quality stratification by reviewer; Table S4: Threshold correlation with quality analysis.
