# Supplementary material for: Quantifiable Measures of Abdominal Wall Motion for Quality Assessment of Cine-MRI Slices in Detection of Abdominal Adhesions
Source: J Imaging. 2023 Apr 30;9(5):92. doi: 10.3390/jimaging9050092 (PMC10219278; doi:10.3390/jimaging9050092)
Supplement: Supplementary file 1 [file jimaging-09-00092-s001.zip › jimaging-2351539 -Supplementary Materials.pdf]

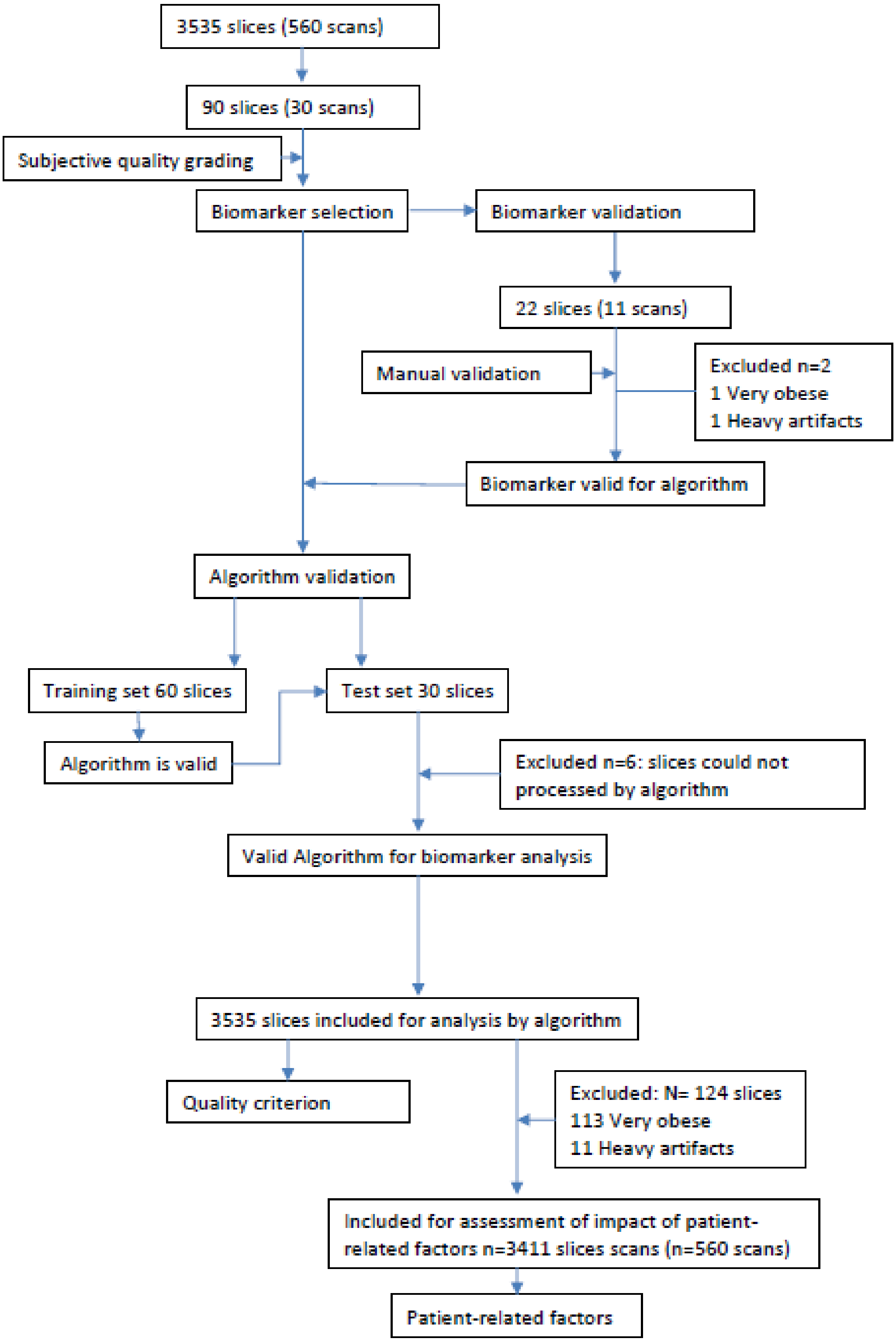

Figure S1. Flowchart Quality Criterion.

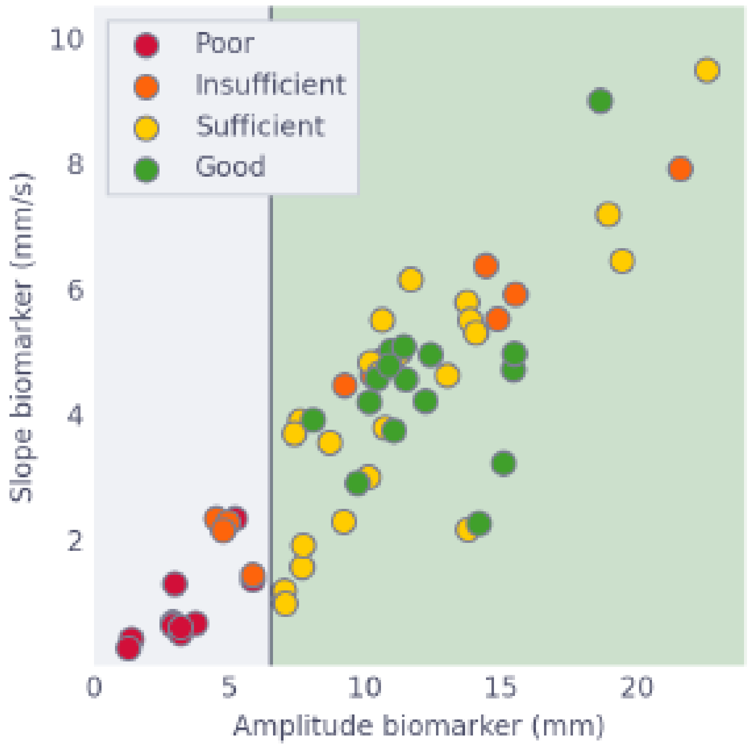

Figure S2. The slope biomarker was excluded from the quality criterion for a few reasons. First, the slope value was not measured manually on the 22 slices on which amplitude and frequency were validated (Figure S1). The reason is that it is much harder to evaluate the maximum slope than the amplitude and frequency, manually. Second, the slope biomarker is heavily correlated (R = 0.88) to the amplitude biomarker, as shown in the Figure above. Third, adding slope to the quality criterion does not result in improved sensitivity or specificity, as visible in the Figure above by the fact that adding a cutoff for slope would only add false positive findings.

Table S1. Correlation of quality assessment between reviewers.

**Intra-Class Coefficient**	**ICC**	**CL 95%**	***p*-value**	**Cronbach's Alpha Value**	
**Overall Quality**				Reviewer 1	
ICC absolute agreement	0,729	0,590-0,821	0.000	Overall	0,818
ICC consistency	0,732	0,593-0,824	0.000	Amplitude	0,752
Kappa	0,256		0.000	Slope	0,725
Amplitude				Frequency	0,769
ICC absolute agreement	0,699	0,513-0,810	0.000	Reviewer 2	
ICC consistency	0,728	0,586-0,821	0.000	Overall	0,875
Kappa	0,233		0.000	Amplitude	0,866
**Slope**				Slope	0,746
ICC absolute agreement	0,683	0,518-0,791	0.000	Frequency	0,847
ICC consistency	0,682	0,516-0,790	0.000	Combined:	
Kappa	0,117		0.029	Overall	0,883
**Frequency**				Amplitude R1	0,856
ICC absolute agreement	0,634	0,443-0,759	0.000	Slope R1	0,868
ICC consistency	0,632	0,442-0,758	0.000	Frequency R1	0,881
Kappa	0,284		0.000	Amplitude R2	0,869
Sufficient vs Insufficient	0,794	0,708-0,858	0.000	Slope R2	0,839
				Frequency R2	0,859

Table S2. Correlation of biomarker and semi-quantitative quality assessment.

Cine-MRI Overall quality	0 (*n* = 16)	1 (*n* = 22)	2 (*n* = 31)	3 (*n*-21)	*p*-value
Amplitude	2,98 (1,14-6,27)	9,15 (2,04-21,60)	10,68 (5,62-22,56)	12,41 (8,07-22,56)	<0.001
Frequency	0,23 (0,08-0,58)	0,23 (0,4-0,47)	0,25 (0,4-0,38)	0,20 (0,04-0,32)	0.039
Slope	0,69 (0,27-2,45)	4,24 (0,25-7,91)	3,86 (0,99-9,49)	4,58 (1,63-9,01)	<0.001
Unreadable slices by the algorithm	1 (6,3%)	1 (4,5%)	1 (3,3%)	0 (0%)	
Quality per Biomarker					
Quality of Amplitude	0 (*n* = 17)	1 (*n*=22)	2 (*n*=24)	3 (27)	*p*-value
Amplitude	3,03 (1,14-6,27)	10,24 (2,04-21,60)	10,73 (5,62-20,46)	11,66 (6,78-22,56)	<0.001
Unreadable slices	0 (0%)	5 (22,7%)	1 (4,2%)	0 (0%)	
Quality of Frequency	0 (*n*=13)	1 (*n*=29)	2 (*n*=48)	3 (*n*=0)	*p*-value
Frequency	0,23 (0,14-0,58)	0,19 (0,04-0,31)	0,25 (0,13-0,47)		0.045
Unreadable slices	0 (0%)	4 (13,8%)	2 (4,2%)		
Quality of Slope	0 (*n*=15)	1 (*n*=12)	2 (*n*=44)	3 (*n*=19)	*p*-value
Slope	0,70 (0,25-2,45)	3,04 (0,42-6,46)	3,73 (0,85-9,49)	4,79 (2,27-9,01)	<0.001
Unreadable slices	0 (0%)	0 (0%)	0 (0%)	0 (0%)	

Table S3. Biomarker vs Quality stratification by reviewer.

Quality grading Reviewer 1 (median, range)	0 (*n*=9)	1 (*n*=15)	2 (*n*=21)	3 (*n*=36)	4 (*n*=3)	*p*-value
Amplitude	2,97 (1,14-5,84)	5,19 (1,28-13,75)	9,02 (4,77-20,46)	13,52 (6,78-22,56)	11,49 (1,14-22,56)	0,001
	0 (*n* = 7)	1 (*n* = 15)	2 (*n* = 36)	3 (*n*=25)	4 (*n*=1)	
Slope	0,65 (0,27-2,45)	2,35 (0,25-6,15)	4,11 (0,42-9,49)	4,79 (0,99-9,01)	4,22 (4,22-4,22)	0,001
	0 (*n* = 13)	1 (*n* = 14)	2 (*n* = 7)	3 (*n* = 1)		
Frequency	0,19 (0,04-0,58)	0,21 (0,04-0,47)	0,21 (0,04-0,47)	0,37 (0,37-0,37		0,52
Total quality grading Reviewer 1	0 (*n*=14)	1 (*n*=11)	2 (*n*=27)	3 (*n*=30)	4 (*n*=2)	*p*-value
Amplitude	2,95 (1,14-6,27)	5,91 (2,04-20,46)	10,17 (4,77-19,24)	13,51 (7,04-22,56)	11,86 (11,49-12,23)	0,001
	0 (*n*=14)	1 (*n*=11)	2 (*n*=27)	3 (*n*=30)	4 (*n*=2)	
Slope	0,68 (0,27-2,45)	2,35 (0,25-5,93)	3,91 (1,21-6,60)	4,87 (0,99-9,49)	4,39 (4,22-4,56)	0,001
	0 (*n*=14)	1 (*n*=11	2 (*n*=27)			
Frequency	0,24 (0,04-0,58)	0,17 (0,04-0,47)	0,23 (0,04-0,38)	0,20 (0,04-0,32)	0,22 (0,20-0,24)	0,394
Quality grading Reviewer 2	0 (*n*=23)	1 (*n*=16)	2 (*n*=9)	3 (*n*=33)	4 (*n*=3)	*p*-value
Amplitude	3,75 (1,14-21,60)	10,60 (2,04-18,95)	8,67 (5,62-14,07)	11,06 (9,21-19,24)	10,21 (1,14-22,56)	0,01
	0 (*n*=17)	1 (*n*=8)	2 (*n*=10)	3 (*n*=49)		
Slope	0,69 (0,25-2,88)	3,54 (0,99-6,46)	3,86 (1,38-5,93)	4,58 (1,21-9,49)		0,001
	0 (*n*=16)	1 (*n*=11	2 (*n*=55)	3 (*n*=1)	4 (*n*=1)	*p*-value
Frequency	0,23 (0,08-0,58)	0,21 (0,10-0,31)	0,23 (0,04-0,38)	0,25 (0,25-0,25)	0,47 (0,47-0,47)	0,586
Total Quality grading Reviewer 2	0 (*n*=15)	1 (*n*=22)	2 (*n*=12)	3 (*n*=32)	4 (*n*=3)	*p*-value
Amplitude	3,03 (1,14-21,60)	10,15 (1,28-22,56)	10,73 (5,62-14,07)	10,91 (5,87-20,56)	16,31 (15,45-19,24)	0,001
	0 (*n*=15)	1 (*n*=22)	2 (*n*=12)	3 (*n*=32	4 (*n*=3)	*p*-value
Slope	0,70 (0,27-7,91)	4,64 (0,25-9,49)	4,20 (0,99-5,86)	4,21 (1,21-9,01)	1,96 (1,63-4,72)	0,026
	0 (*n*=15)	1 (*n*=22)	2 (*n*=12)	3 (*n*=32)	4 (*n*=3)	*p*-value
Frequency	0,25 (0,08-0,58)	0,23 (0,15-0,47)	0,25 (0,10-0,38)	0,20 (0,04-0,37)	0,04 (0,04-0,04)	0,105
Quality grading of both Reviewers						
Quality score	0 (*n*=15)	1 (*n*=18)	2 (*n*=30)	3 (*n*=21)		*p*-value
Amplitude	2,98 (1,14-6,27)	9,15 (2,04-21,60	10,68 (5,62-22,56)	12,41 (8,07-20,56)		0,001
Frequency	0,23 (0,08-0,58)	0,23 (0,04-0,47)	0,25 (0,04-0,38)	0,20 (0,04-0,32)		0,051
Slope	0,69 (0,27-2,45)	4,24 (0,25-7,91)	3,86 (0,99-9,49)	4,58 (1,63-9,01)		0,001
Amplitude Q-both	0 (*n*=17)	1 (*n*=17)	2 (*n*=23)	3 (*n*= 27		*p*-value
Amplitude	3,03 (1,14-6,27)	10,24 (2,04-21,60)	10,73 (5,62-20,46)	11,66 (6,78-22,56)		0,001
Frequency Q-both	0 (*n*=13	1 (*n*=25)	2 (*n*=46)	3 (*n*=0)		*p*-value
Frequency	0,23 (0,14-0,58)	0,19 (0,04-0,31)	0,25 (0,13-0,47)			0,029
Regularity Q-both	0 (*n*=14)	1 (*n*=11)	2 (*n*=40)	3 (*n*=19)		*p*-value
Slope	0,68 (0,25-2,45)	2,88 (0,42-6,46)	3,91 (0,99-9,49)	4,79 (2,27-9,01)		0,001
Sufficient slices	Yes (*n*=51)	No (*n*=33)	*p*-value			
Amplitude	11,22 (5,62-22,56)	5,19 (1,14-21,60	0,001			
Frequency	0,21 (0,04-0,38)	0,23 (0,04-0,58)	0,372			
Slope	4,56 (0,99-9,49)	2,29 (0,25-7,91)	0,007			

Table S4. Threshold correlation with quality analysis.

Pearson's R	Quality R	Quality F	Quality comb	*p*-value
Amplitude >6.5	0,746	0,631	0,751	0.000
Frequency <0.4	0,338	0,329	0,345	0.002
Freq_Ampli	0,749	0,648	0,765	0.000
